# Longitudinal changes in NT-proBNP after breast cancer radiotherapy: clinical determinants of its trajectory and association with cardiac dysfunction (BACCARAT study)

**DOI:** 10.1016/j.ctro.2026.101213

**Published:** 2026-06-03

**Authors:** Médéa Locquet, Gaëlle Jimenez, Jérémy Camilleri, David Broggio, Olivier Lairez, Loïc Panh, Georges Tarlet, Jean Ferrières, Fabien Milliat, Sophie Jacob

**Affiliations:** a Public Health Aging Research and Epidemiology (PHARE) Group, Research Unit in Clinical Pharmacology and Toxicology (URPC), Department of Biomedical Sciences, NAmur Research Institute for LIfe Sciences (NARILIS), Faculty of Medicine, University of Namur, Namur, Belgium; b Department of Radiation Oncology (Orion), Clinique Pasteur, Toulouse, France; c PSE-SANTE/SDOS, Department of Dosimetry, Authority for Nuclear Safety and Radiation Protection (ASNR), Fontenay-aux-Roses, France; d Department of Cardiology, Toulouse University Hospital, Toulouse, France; e Medical School, Toulouse III Paul Sabatier University, Toulouse, France; f Department of Cardiology, Clinique Pasteur, Toulouse, France; g PSE-SANTE/SERAMED/LRMed, Laboratory of Radiobiology of Medical Exposures, Authority for Nuclear Safety and Radiation Protection (ASNR), Fontenay-aux-Roses, France; h National Institute for Medical Research (Inserm), UMR 1295 (CERPOP), Toulouse 31400, France; i Department of Cardiology, Rangueil University Hospital, Toulouse, France; j PSE-SANTE/SESANE/LEPID, Laboratory of Epidemiology, Authority for Nuclear Safety and Radiation Protection (ASNR), Fontenay-aux-Roses, France

**Keywords:** Breast cancer, Radiotherapy, NT-proBNP, Cardiac biomarker, Cancer therapy-related cardiac dysfunction (CTRCD), Cardio-oncology, Radiation-induced cardiotoxicity

## Abstract

**Background and purpose:**

Radiotherapy (RT) for breast cancer (BC) may induce subclinical myocardial injury. Longitudinal trajectories of N-terminal pro-B-type natriuretic peptide (NT-proBNP), a biomarker of myocardial stress, remain insufficiently characterized. We aimed to assess changes in NT-proBNP over 2 years after RT, identify clinical and treatment-associated factors, and evaluate associations with cancer therapy–related cardiac dysfunction (CTRCD).

**Methods:**

NT-proBNP was measured before RT, at RT completion, and at 6 and 24 months among 101 BC patients treated with adjuvant RT without chemotherapy. Echocardiography was performed longitudinally, and CTRCD was defined according to 2022 ESC Cardio-Oncology guidelines. Factors associated with NT-proBNP trajectories were analyzed using linear mixed-effects models. Logistic regression assessed associations between NT-proBNP and CTRCD.

**Results:**

NT-proBNP remained stable at the end of RT and at 6 months but increased at 24 months (β = 0.147, p < 0.0001; +16%). Baseline NT-proBNP was strongly associated with follow-up levels. Hypercholesterolemia (β = 0.252, p = 0.01), slightly moderate hypofractionated RT (20 × 2.35 Gy; β = 0.372, p = 0.0008), and aromatase inhibitor therapy (β = 0.258, p = 0.019) were independently associated with higher NT-proBNP at 24 months, whereas cardiac dose metrics were not. Seventeen patients developed CTRCD. NT-proBNP > 400 ng/L at 24 months was associated with CTRCD (OR = 6.5, 95% CI 1.24–33.85; p = 0.027).

**Conclusion:**

NT-proBNP increased 2 years after breast RT and was associated with baseline level, cardiometabolic, RT fractionation, and endocrine factors. Elevated NT-proBNP could help to identify BC patients at higher risk of subclinical cardiac dysfunction, supporting longitudinal biomarker monitoring in cardio-oncology surveillance.

## Highlights

• Delayed NT-proBNP increase observed 24 months after breast RT.
• Baseline NT-proBNP was associated with longitudinal trajectories.
• Hypercholesterolemia, moderate hypofractionation, and aromatase inhibitors were associated with greater increases.
• Higher NT-proBNP levels were associated with asymptomatic cardiac dysfunction.

## Introduction

Advances in breast cancer (BC) have led to substantial improvements in survival, resulting in a growing population of long-term survivors. Radiotherapy (RT) remains a cornerstone of adjuvant treatment, significantly reducing local recurrence and BC-specific mortality [1]. However, exposure of cardiac structures to ionizing radiation, particularly in left-sided BC, has raised concerns regarding late cardiovascular toxicity [2]. Even with modern RT techniques, radiation-induced cardiac injury can develop years after treatment [3] and often remains subclinical [4], [5], underscoring the need for sensitive tools to detect early myocardial stress.

Cardiac biomarkers have emerged to detect subclinical cardiotoxicity [6], [7], [8]. Among them, N-terminal pro-B-type natriuretic peptide (NT-proBNP) is released by ventricular cardiomyocytes in response to increased wall stress and is widely used for evaluating suspected heart damage, although levels are influenced by non-cardiac factors, in general and oncology populations [7], [9]. In investigations of cardiac dysfunction, elevations in NT-proBNP have been reported after RT. With a median follow-up of 40 months, a prior study found an association between subclinical cardiotoxicity, defined by NT-proBNP level >300 ng/L, and left main coronary artery (LMCA) doses in BC survivors [10]. Another study demonstrated that NT-proBNP levels were significantly higher 5–22 months after RT *vs*. pre-RT controls, and elevated NT-proBNP levels were correlated with higher radiation doses to small cardiac and ventricular volumes [11]. Conversely, Chufal *et al.* showed that 3 months post-RT, NT-proBNP did not differ from pre-RT and was not associated with the mean heart dose [12].

Most available data are cross-sectional or limited to short-term follow-up, providing an incomplete picture of NT-proBNP trajectories at the long term after RT It remains unclear whether transient increases normalize, whether persistent elevations identify patients at higher risk of CTRCD, or how clinical and treatment-related factors are associated with trajectories.

The BACCARAT study was designed as a prospective cohort to investigate the longitudinal evolution of NT-proBNP, among others, in patients treated with BC RT, as a marker of subclinical cardiac changes [12]. In this analysis, we aimed to (1) characterize longitudinal NT-proBNP changes from baseline to 24 months, (2) identify clinical and treatment-related factors, and (3) assess the association between NT-proBNP and CTRCD.

## Methods

### Design and population

This observational study was conducted within the BACCARAT cohort (*BreAst Cancer and CArdiotoxicity Induced by RAdioTherapy*; ClinicalTrials.gov NCT02605512) [13], a prospective cohort designed to investigate subclinical cardiac changes during adjuvant BC RT. The sample size was determined based on the predefined primary endpoint, consisting of a decrease in global or segmental longitudinal strain or strain rate of ≥ 5% and/or an increase of ≥ 15% in the number of coronary segments containing any plaque, and the expected number of eligible patients treated at the study center during the planned inclusion period [13] The present analysis was conducted as an exploratory ancillary study on collected blood samples. In brief, this prospective single-center study enrolled 113 women aged 40–75 years with unilateral (left- or right-sided) BC treated with adjuvant RT following surgical treatment (breast-conserving surgery or mastectomy) at Clinique Pasteur (Toulouse, France) between October 2015 and December 2017. None of the patients received chemotherapy. Exclusion criteria included metastatic disease, prior thoracic irradiation, history of cardiovascular disease, renal insufficiency, and pregnancy. Participants were recruited at baseline (before initiation of RT) and followed longitudinally for 2 years, with cardiac imaging, including echocardiography, and repeated blood sample collections. Of the initial cohort, 5 patients withdrew consent, and 12 had incomplete data (missing dosimetry, biomarker, or echocardiography measurements). Thus, the final study population comprised 101 patients.

The study received ethical approval from the French Southwest Committee for Protection of Persons (CPP2015/66/2015-A00990-69) and the National Agency for Medical and Health Product Safety (Reference: 150873B-12). All participants provided written informed consent.

### Radiotherapy

All BC patients received 3D conformal radiotherapy (3D-CRT) after breast-conserving surgery or mastectomy surgery. Regional lymph nodes (supraclavicular and/or internal mammary) were irradiated when indicated. The standard prescribed dose was 50 Gy in 25 daily fractions (2 Gy per fraction). Due to temporary technical constraints (limited machine availability), 22 patients received moderate hypofractionation (47 Gy in 20 fractions of 2.35 Gy, over 5 weeks).

Treatment was mainly delivered using 6 MV photons, with 25 MV photons used in selected cases with larger breast volumes. A tumor bed boost of 9–16 Gy was delivered using electron or photon beams (6–18 MeV), according to individual anatomy. Dose calculation, including cardiac dosimetry, was performed using the Eclipse™ Treatment Planning System with the Analytical Anisotropic Algorithm (AAA v13.6, Varian Medical Systems). Treatment planning followed ICRU recommendations, and QUANTEC constraints were applied to organs at risk, particularly the heart. Deep inspiration breath hold (DIBH) was selectively used for left-sided BC to meet cardiac constraints (mean heart dose <5 Gy; V25 < 10%).

### Cardiac dosimetry

Radiation dose to the whole heart and cardiac substructures was assessed using individualized dosimetry based on treatment planning CT. Cardiac contours, including the whole heart, left ventricle (LV), and left anterior descending coronary artery (LAD), were delineated according to the previously published BACCARAT methodology. [13], [14]. Dose-volume histograms (DVHs) were generated from the 3D dose matrix. For each structure, the following dosimetry metrics were extracted: mean dose (Dmean, Gy), near-maximum dose to 2% of the volume (D2, Gy), and the percentage volume receiving ≥2 Gy (V2, %).

### NT-proBNP measurement

Blood samples were collected into EDTA-containing tubes, centrifuged, and stored at −80°C until analysis. They were obtained before RT (V0), at the end of RT (V1), 6 months after the end of RT (V6), and 24 months after the end of RT (V24). Plasma NT-proBNP was measured by ELISA (Abnova; Bio-Techne). The levels of all these analytes were measured with the microplate reader system TECAN Infinite M200 Pro, using Magellan software, and concentrations were calculated from a multi-point calibration curve using 4-parameter logistic regression (myassays.com). All samples were analyzed in duplicate, and the mean value was used for statistical analyses. Based on manufacturer recommendations for DuoSet ELISA performance and internal assay conditions, the intra-assay and inter-assay coefficients of variation for NT-proBNP measurements were <10% and <15%, respectively. Quality control samples were included on each plate to monitor assay performance. Finally, NT-proBNP measurements in ng/L were available at V0 (n = 101 patients), V1 (n = 99 patients), V6 (n = 100 patients), and V24 (n = 95 patients). For exploratory purposes, a threshold of NT-proBNP >400 ng/L was used based on heart failure guidelines from ESC recommendations [9].

### Cardiac imaging and definition of cancer therapy-related cardiac dysfunction (CTRCD)

Two-dimensional speckle-tracking echocardiography was performed at baseline and during follow-up. Left ventricular ejection fraction (LVEF) was determined using Simpson’s biplane method [15] and global longitudinal strain (GLS) was determined as the level of deformation between systole and diastole, expressed as a percentage [16]. Asymptomatic CTRCD was defined according to 2022 European Society of Cardiology (ESC) Cardio-Oncology guidelines [7], based on changes in LVEF and GLS during follow-up (LVEF reduction to <40%, or LVEF reduction by ≥10% points to an LVEF of 40–49%, or (LVEF reduction by < 10% points to an LVEF of 40–49% and relative decline in GLS by >15% from baseline), or (LVEF ≥50% and new relative decline in GLS by >15% from baseline). The occurrence of CTRCD was evaluated at 24 months post-RT.

### Clinical and treatment-related covariates

Baseline cardiovascular risk factors were collected, including age, body mass index (BMI), smoking status, hypertension, diabetes mellitus, hypercholesterolemia, and glomerular filtration rate. BC treatment characteristics (i.e., surgery type, endocrine therapy, RT fractionation, and laterality) were recorded. Endocrine therapy was categorized as no endocrine therapy, tamoxifen, or aromatase inhibitor therapy.

### Statistics

Continuous variables were summarized as mean ± standard deviation (SD) or median [interquartile range (IQR)], and categorical variables as counts and percentages. NT-proBNP values were log-transformed due to right-skewed distribution. The statistical model was built using a stepwise approach. Longitudinal changes in NT-proBNP were first analyzed using linear mixed-effects models with random participant-level intercepts to account for repeated measures. Time (pre-RT, end of RT, 6 months, and 24 months post-RT), clinical and treatment-related covariates, and their interactions were included as fixed effects, with an unstructured covariance matrix. Time was treated as a categorical variable. Models were adjusted for baseline NT-proBNP. Variables with significant time interactions (p < 0.05) were then included in a multivariable mixed-effects model to identify factors independently associated with NT-proBNP evolution. Results are reported as β coefficients with standard errors (SE) and p-values. Missing data were limited, and linear mixed-effects models allowed inclusion of all available observations under a missing-at-random assumption. Model assumptions were assessed by examining residual distributions and model fit. Correlations between variables, particularly among dosimetry metrics, were considered to avoid collinearity, and only non-redundant variables were retained in the multivariable analysis. To complement longitudinal analyses, we assessed the association between NT-proBNP and cancer therapy-related cardiac dysfunction (CTRCD) at 24 months. A threshold-based approach using NT-proBNP > 400 ng/L was applied. Logistic regressions adjusted for age, body mass index, hypertension, hypercholesterolemia, smoking status, and relevant dosimetry parameters were used to estimate odds ratios (ORs) with 95% confidence intervals (CIs). All tests were two-sided, with p < 0.05 considered statistically significant. Analyses were performed using SAS® version 9.4 (SAS Institute, Cary, NC, USA).

## Results

### Study population

A total of 101 patients were included in this longitudinal analysis. Baseline characteristics are summarized in Table 1. The mean age was 58.2 ± 8.2 years, and 47% were current or former smokers. Hypercholesterolemia was present in 34% of patients, hypertension in 17%, and diabetes in 6%. Most patients received left-sided breast RT (82%), and 22% received a moderate hypofractionation (20 fractions at 2.35 Gy). Median baseline NT-proBNP was 207 ng/L [IQR: 163–242]. Baseline LVEF and GLS were in the normal range (61% and −19.5%, respectively) with no correlation with NT-proBNP (r = 0.08, p = 0.38, and r = -0.009, p = 0.92, respectively). Mean heart dose was 2.51 ± 1.48 Gy, with higher doses for left-sided BC (Supplementary Table 1). Mean dose to the left ventricle was 5.26 ± 3.86 Gy, while LAD exposure was substantially higher (13.17 ± 9.18).

**Table 1:** Baseline clinical, treatment, and dosimetry characteristics of the BACCARAT cohort (N = 101).

	Whole cohort (N = 101)
	Mean ± SD
	Median [interquartile range]
	N (%)
Clinical characteristics	
Age, years	58.2 ± 8.2
BMI, kg/m^2^	24.5 ± 4.3
Current or past smokers	47 (47%)
Hypertension	17 (17%)
Diabetes	6 (6%)
Hypercholesterolemia	34 (34%)
Glomerular filtration rate, mL/min/1.73 m^2^	89.3 ± 12.8
Menopause	70 (70%)
Left Ventricular Ejection Fraction, %	61 [57–66]
Global Longitudinal Strain, %	−19.5 [–22.17−−17.28]
Baseline NT-proBNP, ng/L	207 [163–242]; Min = 82; Max = 1,308
NT-proBNP 6 months post-RT, ng/L	207 [162–239]; Min = 30; Max = 1,258
NT-proBNP 24 months post-RT, ng/L	245 [186–290]; Min = 74; Max = 1,498

Cancer treatment	
Surgery	
*Conservative*	96 (96%)
*Mastectomy*	5 (5%)
Endocrine therapy	
*No*	26 (26%)
*Tamoxifen*	31 (31%)
*Anti-aromatase*	44 (44%)
Delivered dose	
50 Gray (Gy), 25x2Gy	79 (79%)
47 Gy, 20x2.35 Gy (moderate hypofractionated)	22 (22%)
Left-sided BC	82 (82%)
Whole heart dose	
Mean dose, Gy	2.50 ± 1.49
D2, Gy	22.58 ± 18.00
V2, %	24.19 ± 15.32
Left ventricle dose	
Mean dose, Gy	5.26 ± 3.86
D2, Gy	28.77 ± 19.18
V2, %	40.21 ± 22.75
Left anterior descending coronary artery dose	
Mean dose, Gy	13.17 ± 9.18
D2, Gy	31.78 ± 19.42
V2, %	72.05 ± 34.31

BC: breast cancer; BMI: body mass index; RT: radiotherapy; Gy: Gray; SD: standard deviation

### Longitudinal changes of NT-proBNP after radiotherapy

**Fig. 1:**
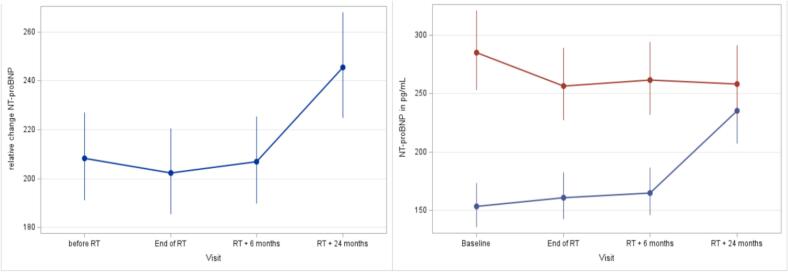
Longitudinal trajectories of NT-proBNP levels before and after radiotherapy (RT). Predicted NT-proBNP levels are shown at baseline (pre-RT), end of RT, 6 months, and 24 months post-RT, based on unadjusted linear mixed-effects models. Points represent least squares (LS) means (predicted values), and error bars 95% confidence intervals (CI). Left panel: overall cohort. Right panel: trajectories stratified by baseline NT-proBNP level: low (< median, 207 ng/L; blue) and high (≥median; red). (For interpretation of the references to colour in this figure legend, the reader is referred to the web version of this article.)

NT-proBNP levels over time are described in Supplementary Fig. 1. In the crude mixed-effects model, NT-proBNP levels did not change significantly at the end of RT or at 6 months. Nevertheless, they increased significantly at 24 months post-RT (β = 0.147, p = 0.0003), corresponding to a 16% relative increase *vs* baseline. The overall time effect was significant (p < 0.0001) (Supplementary Table 2). Baseline NT-proBNP was strongly associated with follow-up values (p < 0.0001). A marginally significant interaction of baseline NT-proBNP with time was observed (p = 0.058), with a significant interaction at 24 months post-RT (β = − 0.00051, p = 0.010), indicating a smaller increase, or even a slight decrease, among patients with higher baseline levels in contrast with the increase observed in patients with lower baseline NT-proBNP (Fig. 1).

### Analysis of potential modifiers of NT-proBNP trajectory

In this exploratory analysis,univariate models adjusted for baseline NT-proBNP (Table 2), age, smoking status, renal function, hypercholesterolemia, endocrine therapy, and radiotherapy fractionation significantly modified NT-proBNP trajectories over time (p for interaction <0.05). No significant associations were observed for hypertension, diabetes, menopausal status, baseline echocardiographic parameters, or cardiac radiation dose metrics. Baseline characteristics and cardiac dose metrics were comparable between fractionation groups (all p < 0.05, data not detailed).

**Table 2:** Predictors of NT-proBNP longitudinal changes after RT: univariate analysis with adjustment on baseline NT-proBNP.

	P-value	β-estimate (Standard error (SE))
Visit	0.148	
Age	0.355	
Age × Visit	**0.037**	
*Age × Visit 6*	0.971	0.0002 (0.0005)
*Age × Visit 24*	**0.023**	0.012 (0.005)
Body mass index	NS	−
Visit	**0.0002**	
Smoking	0.054	
Smoking x Visit	**0.044**	
*Smoking x Visit 6*	0.342	0.081 (0.085)
*Smoking x Visit 24*	0.119	−0.137 (0.088)
Hypertension x Visit	NS	−
Diabetes x Visit	NS	−
Visit	**00,002**	
Glomerular filtration rate (GRF)	**0.076**	
Glomerular filtration rate x Visit	**0.011**	
*GFR x Visit 6*	**0.563**	−0.002 (0.003)
*GFR x Visit 24*	**0.004**	−0.011 (0.004)
Visit	**<0.0001**	
Hypercholesterolemia	**0.005**	
Hypercholesterolemia x Visit	**0.003**	
*Hypercholesterolemia x Visit 6*	0.397	0.046 (0.089)
*Hypercholesterolemia x Visit 24*	**0.001**	0.307 (0.091)
Menopause x Visit	NS	−
Baseline left ventricular ejection fraction x Visit	NS	−
Baseline global longitudinal strain x Visit	NS	−
Surgery x Visit	NS	−
Visit	**0.002**	
Endocrine therapy	0.402	
Endocrine therapy x Visit	**0.004**	
*Tamoxifen*		NS
*Anti-Aromatase x Visit 6*	0.351	0.096 (0.103)
*Anti-Aromatase x Visit 24*	**0.032**	0.230 (0.106)
Visit	0.142	
Hypofractionation	**0.011**	
Hypofractionated x Visit	**0.0004**	
*Hypofractionated x Visit 6*	0.656	−0.044 (0.0100)
*Hypofractionated x Visit 24*	**0.001**	**0.338 (0.102)**
Left-sided breast cancer x Visit	NS	
Whole heart dose		
Mean dose x Visit	NS	−
D2 x Visit	NS	−
V2 x Visit	NS	−
Left ventricle dose		
Mean dose x Visit	NS	−
D2 x Visit	NS	−
V2 x Visit	NS	−

NS: not significant (p > 0.05); β coefficients, standard errors (SEs), and 95% confidence intervals (CIs) were estimated using linear mixed-effects models on log-transformed NT-proBNP;D2 (Gy): near-maximum dose (dose received by 2% of the organ volume), V2 (%): volume receiving ≥ 2 Gy (percentage of organ volume); P-values in bold for p < 0.05.

**Fig. 2:**
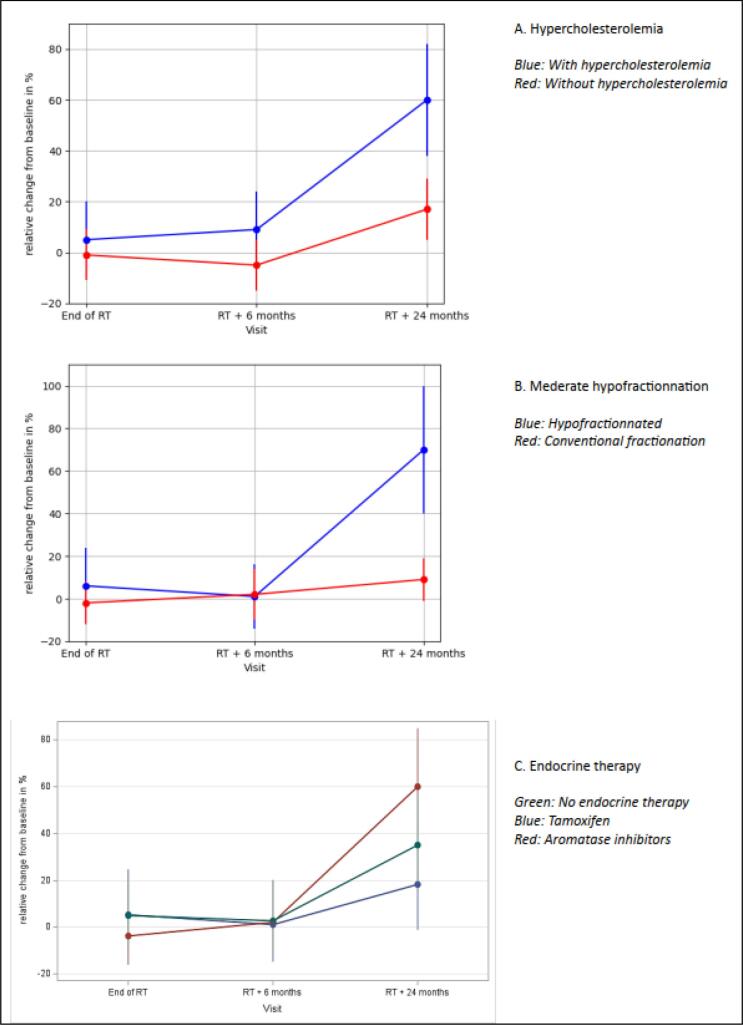
Predicted percentage changes in NT-proBNP from baseline according to selected factors in breast cancer patients treated with radiotherapy (RT). Predicted relative changes (%) in NT-proBNP from baseline are shown at the end of RT, 6 months, and 24 months post-RT, after adjustments. Points represent least squares (LS) means, and error bars 95% confidence intervals (CI). (A) Stratification by hypercholesterolemia (blue: with; red: without). (B) Stratification by RT fractionation (blue: moderate hypofractionation; red: conventional fractionation). (C) Stratification by endocrine therapy (green: none; blue: tamoxifen; red: aromatase inhibitors). (For interpretation of the references to colour in this figure legend, the reader is referred to the web version of this article.)

In the multivariate mixed-effects model, including variables with p < 0.05 in the univariate analysis (Table 3), baseline NT-proBNP remained strongly associated with longitudinal NT-proBNP levels (p = 0.013). Hypercholesterolemia and slightly moderate hypofractionation remained associated with higher NT-proBNP levels over time, with a significant interaction at 24 months post-RT (β for hypercholesterolemia = 0.252, p = 0.010; β for moderate hypofractionation = 0.372, p < 0.001). At 24 months, patients with hypercholesterolemia showed an approximately 60% increase in NT-proBNP, compared with a 17% increase in patients without hypercholesterolemia, and patients who received moderate hypofractionated RT showed an approximately 71% increase in NT-proBNP *vs* 9% increase in patients who received conventional fractionation (Fig. 2). Endocrine therapy showed a borderline interaction with time (p = 0.054), driven by higher NT-proBNP levels at 24 months post-RT in BC patients receiving aromatase inhibitors, corresponding to approximately a 60% increase in NT-proBNP *vs.* 18% in patients receiving tamoxifen or 34% in patients receiving no endocrine therapy (Fig. 2).

**Table 3:** Predictors of NT-proBNP longitudinal changes after radiotherapy (RT): multivariate analysis with adjustment on baseline NT-proBNP.

	P-value	β-estimate (Standard error (SE))
Baseline NT-proBNP	**0.013**	−0.0004 (0.0002)
Visit	0.358	
Age	0.294	
Age x Visit	0.848	
Smoking	0.865	
Smoking x Visit	0.411	
Glomerular filtration rate (GFR)	0.263	
GFR x Visit	0.573	
Hypercholesterolemia	**0.004**	
Hypercholesterolemia x Visit	**0.031**	
*Hypercholesterolemia x Visit 24*	**0.010**	0.252 (0.096)
Moderate hypofractionated	**0.011**	
Moderate hypofractionated x Visit	**<0.0001**	
*Moderate hypofractionated x Visit 24*	**0.0008**	0.372 (0.108)
Endocrine therapy	0.682	
Endocrine therapy x Visit	0.054	
*Anti-Aromatase x Visit 24*	**0.019**	0.258 (0.109)

NS: not significant (p > 0.05); β coefficients and standard error were estimated using linear mixed-effects models on log-transformed NT-proBNP. P-values in bold for p < 0.05.

### Threshold-based analysis of NT-proBNP levels and association with CTRCD

According to the ESC guidelines [7], 17 patients developed CTRCD 24 months after RT. Baseline clinical characteristics were comparable between BC patients with and without CTRCD (all p < 0.05). Higher baseline NT-proBNP was significantly associated with higher risk of CTRCD (OR per 10 ng/L = 1.03, p = 0.048) (Table 4) whereas non-significant trend for higher risk of CTRCD with higher level of NT-ProBNP at follow-up measurement was observed. Using an NT-proBNP cutoff of 400 ng/L, the only significant association with CTRCD was for NT-proBNP at 24 months: 10 patients exceeded 400 ng/L and 4 (40%) had CTRCD simultaneously, compared with 15% of patients with NT-proBNP ≤ 400 ng/L (p = 0.048). In adjusted logistic regression association between NT-proBNP > 400 ng/L at 24 months post-RT and CTRCD remained significant (OR = 6.5, 95% CI: 1.24–33.85, p = 0.027) (Table 4).

**Table 4:** Association between NT-proBNP and cancer-therapy related cardiac dysfunction (CTRCD).

	OR* (95%CI)	P-value
**Continuous NT-proBNP, ng/L**		
Baseline	Per 10 ng/L: 1.03 (1.00–1.05)	**0.048**
End of RT	Per 10 ng/L: 1.02 (0.99–1.05)	0.056
6 months post-RT	Per 10 ng/L: 1.03 (0.99–1.06)	0.064
24 months post-RT	Per 10 ng/L: 1.02 (1.00–1.05)	0.079

**NT-proBNP > 400 ng/L**		
Baseline (n = 7)	3.66 (0.46–29.24)	0.219
End of RT (n = 6)	3.29 (0.41–26.46)	0.292
6 months post-RT (n = 7)	3.29 (0.41–26.46)	0.292
24 months post-RT (n = 10)	6.39 (1.20–34.07)	**0.029**

Abbreviations: OR: odds ratio; CI: confidence interval; IQR: interquartile range; RT: radiotherapy; CTRCD: cancer-therapy-related cardiac dysfunction; V24: visit at 24 months after RT.

*Adjusted for age, BMI, hypertension, smoking, hypercholesterolemia, endocrine therapy, and mean circumflex coronary dose.

Finally, we present clinical and treatment-related characteristics and echocardiographic parameters used to define CTRCD for the 7 patients who exceeded 400 ng/L at baseline, of whom 5 remained above the threshold at visit 24 months (Supplementary Table 3). Moreover, 5 patients newly crossed the NT-proBNP threshold at 24 months post-RT (Supplementary Table 4).

## Discussion

In this 2-year study, NT-proBNP showed a delayed increase after BC RT, with trajectories associated with baseline levels. Hypercholesterolemia, moderate hypofractionated RT, and endocrine therapy, particularly aromatase inhibitor use, were associated with NT-proBNP trajectories. NT-proBNP > 400 ng/L at 24 months was associated with cardiac dysfunction (CTRCD). Overall, NT-proBNP levels remained stable at the end of RT and at 6 months but increased significantly at 24 months post-RT. Baseline NT-proBNP emerged as a key correlate of subsequent trajectory: patients with lower baseline levels showed greater relative increases, whereas those with higher baseline values remained persistently elevated. This interaction can reflect differing myocardial reserve or ceiling effects, consistent with NT-proBNP's role as a marker of myocardial vulnerability [17]. Failure to account for baseline values can hide real longitudinal changes and lead to misleading conclusions due to regression toward the mean. By adjusting for baseline NT-proBNP and modeling time-dependent interactions, our study provides a more accurate assessment of dynamics.

We did not observe associations between changes in NT-proBNP and cardiac radiation dose. This may reflect the limitations of conventional dose-volume metrics, as the spatial distribution of radiation within cardiac substructures may be more relevant than mean heart dose alone. Previous studies investigating the association between NT-proBNP and heart dose after breast RT have reported inconsistent findings, with some identifying associations with specific dose-volume parameters [18], while others found no significant association [10], [18], [19]. Our findings are therefore consistent, suggesting that factors other than global dose-volume metrics could play a more important role in cardiac biomarker changes [12].

Hypercholesterolemia emerged as a potential modifier of NT-proBNP trajectories, with patients showing a substantially greater increase at 24 months post-RT (approximately 60% *vs* 17% in those without hypercholesterolemia). Dyslipidemia is known to contribute to endothelial dysfunction and microvascular disease, which may enhance susceptibility to radiation-induced cardiac injury [20], [21]. In our study, hypercholesterolemia was associated with a higher risk of CTRCD, although the association was not significant (OR = 1.9, p = 0.28). Hypercholesterolemia may also be associated with coronary artery calcification progression, consistent with underlying atherosclerosis, as previously reported in this cohort [22].

Endocrine therapy showed a borderline association with NT-proBNP trajectory, with higher levels at 24 months, consistent with prior evidence[23]. However, the use of aromatase inhibitors was not associated with a higher risk of CTRCD in our analysis.

Moderate hypofractionated RT was also associated with a higher increase in NT-proBNP at 24 months (approximately 71% *vs.* 9% with conventional fractionation), despite comparable mean heart doses. Evidence regarding fractionation and cardiac biomarkers remains limited, although experimental data suggest that cardiac tissue is sensitive to fraction size [24]. In our study, moderate hypofractionation was associated with higher NT-proBNP levels at 24 months, despite comparable mean heart doses. Although its association with CTRCD did not reach statistical significance, other prospective studies also reported signals of subclinical myocardial injury with higher doses per fraction [25], [26]. These findings support further investigation of RT fractionation in cardio-oncology. Importantly, at baseline, LVEF, GLS, and NT-proBNP were within normal ranges, and no patient developed symptomatic heart failure during follow-up. The cohort, therefore, reflects a population without overt cardiac disease, and observed alterations were predominantly early subclinical cardiac changes (17 patients with asymptomatic CTRCD at 24 months). Higher baseline NT-proBNP was associated with increased CTRCD risk, underscoring the importance of pre-treatment myocardial vulnerability. Using a threshold of 400 ng/L, proposed by NICE heart failure guidelines [27], we showed that NT-proBNP levels >400 ng/L at 24 months identified a subgroup at higher risk of CTRCD. This threshold was applied for exploratory purposes in an asymptomatic population. Although NT-proBNP cutoffs are limited by the effects of aging, renal function, BMI, and other comorbidities [9], crossing this threshold was associated with a more than 6-fold increase in the odds of CTRCD. Patients with elevated baseline NT-proBNP tended to remain above this threshold, indicating sustained vulnerability. Conversely, patients with initially low NT-proBNP who developed significant increases over time may represent a clinically actionable subgroup. Notably, half of the patients with 400 ng/L or higher at 24 months had newly developed elevations, and 40% showed systolic dysfunction. These findings support the potential value of NT-proBNP as a dynamic marker of late subclinical cardiac changes after breast RT.

Distinguishing treatment-related cardiotoxicity from evolving cardiovascular risk remains challenging in cardio-oncology. NT-proBNP may increase over time with aging and cardiometabolic changes. Specifically, a population-based study showed that NT-proBNP tends to increase over time with aging and evolving cardiovascular risk, and that its delayed dynamic changes reflected changes in cardiovascular risk [28]. In our study, the delayed increase observed at 24 months was associated with baseline myocardial vulnerability, cardiovascular risk factors, endocrine therapy, and RT characteristics rather than radiation dose metrics.

### Strengths and limitations

The main strength of this study is its prospective longitudinal design with repeated NT-proBNP assessments from baseline through 24 months, combined with imaging and dosimetry. This allowed detailed characterization of biomarker trajectories in RT settings. Limitations should be acknowledged. First, the sample size was moderate, limiting statistical power, particularly for multivariable and interaction analyses, and leading to wide 95% CIs for some associations. Nevertheless, this cohort remains among the largest prospective studies to assess serial biomarkers and imaging after breast RT. Second, attrition could affect our findings, as approximately 11% of BC patients were lost to follow-up. However, baseline characteristics were similar between patients lost to follow-up and those included in the analysis, supporting a limited risk of attrition bias. Third, follow-up was limited to 24 months, limiting differentiation between transient biomarker elevation and progressive cardiac dysfunction. However, the association between NT-proBNP elevation and CTRCD supports its clinical relevance within this timeframe. Fourth, NT-proBNP is influenced by multiple other factors, such as statin use, blood pressure medications, lifestyle factors including physical activity, and cardiometabolic status; residual confounding cannot be excluded despite adjustments. Finally, in the absence of a non-irradiated comparison group, caution is warranted when interpreting the contribution of radiotherapy to the observed changes.

## Conclusion

Delayed NT-proBNP elevation after breast RT may reflect baseline myocardial vulnerability interacting with cardiometabolic risk, endocrine therapy, and possibly fractionation, rather than dose. Longitudinal monitoring could inform individualized cardio-oncology surveillance. Larger studies with longer follow-up are needed to clarify fractionation effects, link biomarker trajectories with imaging, and test whether NT-proBNP-guided strategies improve long-term cardiovascular outcomes in BC survivors.

## Funding source

This study was supported by funding from *Fédération Française de Cardiologie* (FFC), *Electricité de France* (EDF), and the “H2020 Euratom research and training programme 2014–2018” (grant agreement No. 755523).

## Declaration of generative AI and AI-assisted technologies in the writing process

During the preparation of this work, the authors used ChatGPT (OpenAI) to improve language and readability. After using these tools/services, the authors reviewed and edited the content as needed and took full responsibility for the publication's content. These AI tools were not used to generate content.

## CRediT authorship contribution statement

**Médéa Locquet:** Writing – review & editing, Writing – original draft, Visualization, Validation, Software, Resources, Methodology, Investigation, Formal analysis, Data curation, Conceptualization. **Gaëlle Jimenez:** Writing – review & editing, Resources. **Jérémy Camilleri:** Writing – review & editing, Resources. **David Broggio:** Writing – review & editing, Validation, Software, Resources, Methodology. **Olivier Lairez:** Writing – review & editing, Validation, Software, Resources. **Loïc Panh:** Writing – review & editing, Validation, Resources. **Georges Tarlet:** Writing – review & editing, Visualization, Validation, Resources, Methodology. **Jean Ferrières:** Writing – review & editing, Visualization, Validation, Methodology. **Fabien Milliat:** Writing – review & editing, Visualization, Validation, Resources, Methodology. **Sophie Jacob:** Writing – review & editing, Writing – original draft, Visualization, Validation, Supervision, Software, Resources, Project administration, Methodology, Investigation, Funding acquisition, Formal analysis, Data curation, Conceptualization.

## Ethics approval

The French Southwest Committee for Protection of Persons (CPP2015/66/2015-A00990-69) and the National Agency for Medical and Health Product Safety (150873B-12) approved the BACCARAT cohort study.

## Declaration of competing interest

The authors declare that they have no known competing financial interests or personal relationships that could have appeared to influence the work reported in this paper.

## Data Availability

Data are accessible upon reasonable written request to *Autorité de Sureté Nucléaire et Radioprotection* (ASNR) at sophie.jacob@asnr.fr, in compliance with strict institutional policies and the European General Data Protection Regulation (EU Regulation 2016/679).
