# Supplementary material for: Longitudinal changes in NT-proBNP after breast cancer radiotherapy: clinical determinants of its trajectory and association with cardiac dysfunction (BACCARAT study)
Source: Clin Transl Radiat Oncol. 2026 Jun 3;60:101213. doi: 10.1016/j.ctro.2026.101213 (PMC13263734; doi:10.1016/j.ctro.2026.101213)

**Supplementary Tables**

| **Supplementary Table 1. Cardiac dosimetry according to breast cancer (BC) laterality** | | |
| --- | --- | --- |
|  | **Left-sided BC**  **Mean ± SD** | **Right-sided BC**  **Mean ± SD** |
| Whole heart  Mean dose, Gy  D2, Gy  V2, % | 2.91 ± 1.29  26.97 ± 16.93  27.97 ± 13.02 | 0.61 ± 0.46  2.47 ± 1.14  6.77 ± 13.07 |
| Left Ventricle  Mean dose, Gy  D2, Gy  V2, % | 6.38 ± 3.34  35.01 ± 15.25  48.74 ± 14.68 | 0.17 ± 0.26  0.53 ± 0.60  1.04 ± 4.30 |
| Left anterior coronary artery  Mean dose, Gy  D2, Gy  V2, % | 15.98 ± 7.63  38.60 ± 14.04  86.31 ± 13.53 | 0.28 ±0.53  0.51 ± 0.73  6.63 ± 22.29 |

BC: breast cancer; Gy: Gray; SD: standard deviation

| **Supplementary Table 2.** NT-proBNP levels at each visit and mixed-effects model estimates of post-radiotherapy (RT) changes relative to baseline (pre-RT) | | | |  |
| --- | --- | --- | --- | --- |
|  |  | **β-estimate**  **(Standard error (SE))** | **P-value** |  |
| **Crude model** | | | | |
| Pre-RT |  | Reference |  |  |
| End of RT |  | -0.031(0.039) | 0.43 |  |
| 6 months post-RT |  | -0.008 (0.039) | 0.83 |  |
| 24 months post-RT |  | 0.147 (0.040) | **0.0003** |  |
| *Overall time effect* |  |  | **<0.0001** |  |
| **Model including baseline NT-proBNP levels** | | | | |
| Pre-RT |  | Reference |  |  |
| End of RT |  | -0.005 (0.061) | 0.92 |  |
| 6 months post-RT |  | 0.061 (0.062) | 0.32 |  |
| 24 months post-RT |  | 0.275 (0.063) | **<0.0001** |  |
| *Overall time effect* |  |  | **<0.0001** |  |
| Baseline NT-proBNP  Baseline NT-proBNP x Visit  at Visit 24 |  | 0.002 (0.0001)  -  -0.00051 (0.0002) | **<0.0001**  0.058  **0.010** |  |

β coefficients, standard errors (SEs), and 95% confidence intervals (CIs) were estimated using linear mixed-effects models on log-transformed NT-proBNP. P-values in bold for p<0.05.

| **Supplementary Table 3.** Characteristics of breast cancer patients with elevated NT-proBNP (>400 ng/L) at baseline and their longitudinal changes over 24 months post-radiotherapy | | | | | | | | | | | | | | | |  |
| --- | --- | --- | --- | --- | --- | --- | --- | --- | --- | --- | --- | --- | --- | --- | --- | --- |
| **ID** | **Age** | **Breast cancer side** | **Hypercholesterolemia** | **RT fractionation** | **Endocrine therapy** | **LVEF baseline** |  | **LVEF Visit 24** | **GLS baseline** |  | **GLS Visit 24** | **NT-proBNP Visit 0** | **NT-proBNP Visit 1** | **NT-proBNP Visit 6** | **NT-proBNP Visit 24** | **CTRCD at Visit 24** |
| P18 | 53 | Left | No | 25 x 2 Gy | No | 74 % |  | . | -27% |  | . | 495 | 360 | 354 | . | 0 |
| P117 | 53 | Right | No | 25 x 2 Gy | Tamoxifen | 65 % |  | 72 % | -21 % |  | . | 519 | 565 | 430 | 604 | 0 |
| P26 | 59 | Left | Yes | 25 x 2 Gy | Anti-aromatase | 63 % |  | 77 % | -19 % |  | -22 % | 412 | 357 | 689 | 287 | 0 |
| P54 | 56 | Left | No | 20 x 2.35Gy | Tamoxifen | 59 % |  | 65 % | -23 % |  | -23 % | 1,233 | 1,291 | 1,002 | 1,403 | 0 |
| P102 | 66 | Right | No | 25 x 2 Gy | Anti-aromatase | 78 % |  | 89 % | -24% |  | . | 567 | 563 | 528 | 1,050 | 0 |
| P77 | 48 | Left | Yes | 20 x 2.35Gy | No | 72 % |  | 58 % | -24 % |  | **-12 %** | 1,302 | 1,053 | 1,258 | 1,498 | 1 |
| P25 | 64 | Left | Yes | 25 x 2 Gy | No | 55 % |  | **44 %** | -21% |  | -23 % | 1,308 | 1,418 | 1,012 | 956 | 1 |

In grey: patients with NT-ProBNP at Visit 24 > 400 ng/L. In bold: parameters that met Cancer-Therapy-Related Cardiac Dysfunction (CTRCD) definition criteria. Abbreviations: GLS: global longitudinal strain, LVEF: left ventricular ejection fraction, RT: radiotherapy.

| **Supplementary Table 4.** Characteristics of breast cancer patients with **de novo** NT-proBNP (>400 ng/L) and their longitudinal changes over 24 months post-radiotherapy | | | | | | | | | | | | | | | |  |
| --- | --- | --- | --- | --- | --- | --- | --- | --- | --- | --- | --- | --- | --- | --- | --- | --- |
| **ID** | **Age** | **Breast cancer side** | **Hypercholesterolemia** | **RT fractionation** | **Endocrine therapy** | **LVEF baseline** |  | **LVEF Visit 24** | **GLS baseline** |  | **GLS Visit 24** | **NT-proBNP Visit 0** | **NT-proBNP Visit 1** | **NT-proBNP Visit 6** | **NT-proBNP Visit 24** | **CTRCD at Visit 24** |
| P74 | 43 | Left | No | 20 x 2.35Gy | Tamoxifen | 62 % |  | 64 % | -19 % |  | -22 % | 198 | 246 | 226 | 870 | 0 |
| P99 | 67 | Left | Yes | 25 x 2 Gy | Anti-aromatase | 54 % |  | 52 % | -21 % |  | . | 292 | 274 | 338 | 440 | 0 |
| P39 | 62 | Right | No | 25 x 2 Gy | No | 47 % |  | 72 % | -15 % |  | -22 % | 349 | 366 | 350 | 428 | 0 |
| P101 | 56 | Left | Yes | 25 x 2 Gy | Anti-aromatase | 52 % |  | **40 %** | -11 % |  | -13 % | 214 | 202 | 212 | 476 | 1 |
| P68 | 67 | Left | No | 25 x 2 Gy | Anti-aromatase | 60 % |  | **39%** | -14 % |  | -11 % | 337 | 385 | 357 | 700 | 1 |

In bold: parameters that met Cancer-Therapy-Related Cardiac Dysfunction (CTRCD) definition criteria. Abbreviations: GLS: global longitudinal strain, LVEF: left ventricular ejection fraction, RT: radiotherapy.

**Supplementary Figure 1. Longitudinal changes in NT-proBNP levels from pre-radiotherapy to 24 months post-radiotherapy.**NT-proBNP was measured before RT (Visit 0), at the end of RT (Visit 1), and at 6 and 24 months after RT (Visits 6 and 24). Box plots display the distribution of values at each time point, and gray lines indicate individual trajectories over time.
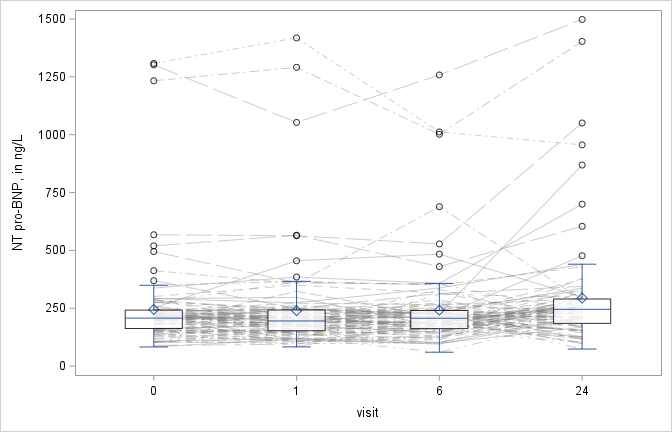

Supplement: Supplementary Data 1 — Supplementary tables and figure. [file mmc1.docx]
